# The trade-off of *Vibrio parahaemolyticus* between bacteriophage resistance and growth competitiveness

**DOI:** 10.3389/fmicb.2024.1346251

**Published:** 2024-06-11

**Authors:** Xiuxiu Zeng, Shanyan Liang, Jiayi Dong, Guosheng Gao, Yaoren Hu, Yuechao Sun

**Affiliations:** ^1^Ningbo No.2 Hospital, Ningbo, Zhejiang, China; ^2^Guoke Ningbo Life Science and Health Industry Research Institute, Ningbo, Zhejiang, China

**Keywords:** vibriophage, bacteriophage therapy, anti-phage mutant, bacteriophage resistance, trade-off

## Abstract

*Vibrio parahaemolyticus* is a food-borne pathogen, which is often isolated from various seafood products. In this study, two kinds of bacteriophages was isolated from the offshore sediments samples. The anti-phage mutant strain were obtained after seventeen rounds of co-culture of *Vibrio parahaemolyticus* and mixed bacteriophage, multigroup sequencing was carried out on spontaneous the anti-phage mutant strain and the wild-type strain. We used the Sanger sequencing to verify the accuracy of the mutation sites. Biolog GEN III MicroPlates were used to evaluate the metabolic capacity of wild-type strains and the anti-phage mutant strain. In this study, we found that with flaG gene (slight homology to N terminus of multiple flagellins) mutated, making the bacteriophage unable to absorb to the cell surface of the host. And, the growth competitiveness of the anti-phage mutant strain is lower than the wild-type strain. These results indicated that the fitness cost, including loss of the growth competitiveness, constitutes a barrier to the prevalence of these defense mechanisms. And the selection pressure on different anti-phage strategies depends on the trade-off between mortality imposed by bacteriophages and fitness cost of the defense strategy under the given environmental conditions. In conclusion, this study provides valuable insights into the phage-host interaction and phage resistance in *Vibrio parahaemolyticus*. Our study provided knowledge for the evolutionary adaption of bacteria against the bacteriophage, which could add more information to understand the phage resistance mechanism before applying in the industry.

## Introduction

*Vibrio parahaemolyticus* is a Gram-negative bacterium, which is widely distributed in seawater, seabed sediments and seafood, eating fresh food containing *Vibrio parahaemolyticus* will cause human infection (Raszl et al., 2016; Kim et al., 2017). Antimicrobial agents have been an important attempt at animal therapy since penicillin was discovered in the 1920s (Aarestrup and Wegener, 1999). In order to control the harm of *Vibrio parahaemolyticus*, antibiotics have to be used in aquaculture. However, antibiotic-resistant *Vibrio parahaemolyticus* appeared because of the overuse of antibiotics and antibiotic-resistant *Vibrio parahaemolyticus* will cause economic losses and threaten human health (Lesmana et al., 2001; Ottaviani et al., 2013; Elmahdi et al., 2016; Kang et al., 2017). Therefore, it is necessary to find a new way to control the spread of antibiotic-resistant *Vibrio parahaemolyticus*.

With the emergence of drug-resistant bacteria, the antibacterial effect of antibiotics has failed and new antibiotics have not been found. Bacteriophage therapy has returned to people’s field of vision because bacteriophage destroys the potential of bacteria (Carlton, 1999; Lu and Collins, 2007, 2009; Sharma et al., 2017; Jamal et al., 2019). The mechanism of bacteriophage therapy is different from that of antibiotics, bacteriolytic bacteriophage can control and kill bacteria in the process of host proliferation (Yang et al., 2019). Importantly, these viruses were nontoxic to humans, bacteriophages cannot survive in the environment alone, and they are aimed at specific host strains (Chan et al., 2018). As early as 1919, bacteriophage therapy treated chickens infected with *Salmonella gallinarum* (Sulakvelidze et al., 2001). Bacteriophage therapy could control bacterial infections in fish, shrimp, and other aquatic products in aquaculture (Matamp and Bhat, 2019; Nikapitiya et al., 2020). Karunasagar et al. (2007) have confirmed that bacteriophage therapy is an effective disease prevention method for aquaculture. Therefore, bacteriophage therapy is an eco-friendly treatment and can effectively kill *Vibrio parahaemolyticus* in aquaculture.

At present, there have been a lot of reports about the isolation and identification of Vibrio bacteriophage and its application in animals (Rong et al., 2014; Alagappan et al., 2016; Raszl et al., 2016; Kalatzis et al., 2018; Ahmmed et al., 2019; Ren et al., 2019; Liang et al., 2022). However, this method has not yet reached the stage of clinical application, and it is still in the exploratory stage (Plaza et al., 2018). Before clinical application, several limiting problems (such as effect, mode of administration, bacteriophage resistance, etc.) must be solved (Kalatzis et al., 2018). In this study, two lytic bacteriophage (PGA and PGB) infecting *Vibrio parahaemolyticus*, was isolated from a large-scale aquaculture areas, and their morphological size, biological characteristics and genetic characteristics were identified. Then the inhibition of PGA and PGB on the growth of *Vibrio parahaemolyticus* were studied and the evolutionary trade-offs between bacteriophage and bacteria were studied. This identification and analysis will deepen our understanding of Vibrio bacteriophage and provide a theoretical basis for controlling *Vibrio parahaemolyticus*.

## Materials and methods

### Sample acquisition

A total of 10 bacterial strains were used in this study (Table 1). A strain of *Vibrio parahaemolyticus* (MCCC 1A16298) came from China Marine Culture Collection Center (MCCC). Other strains were provided by Third Institute of Oceanography of China State Oceanic Administration. All the bacteria were grown in 2216E liquid medium at 28°C.

**Table 1:** Host range of bacteriophage vB_VpaS_PGA and vB_VpaS_PGB.

Strains	Lytic activity of bacteriophage vB_VpaS_PGA	Lytic activity of bacteriophage vB_VpaS_PGB
Bacillus marisflavi	**−**	**−**
Bacillus vietnamensis	**−**	**−**
Pseudomonas xanthomarina	**−**	**−**
Vibrio maritimus	**−**	**−**
Vibrio neocaledonicus	**−**	**−**
Bacillus cereus	**−**	**−**
Bacillus fengqiuensis	**−**	**−**
Bacillus idriensis	**−**	**−**
Oceanbacillus	**−**	**−**
*Vibrio parahaemolyticus*	+++	+++

− No lytic ability; + weak lytic ability; ++ lytic ability; +++ highly strong lytic ability.

### Isolation and purification

The offshore sedimentse (100 g) was collected from an aquaculture areas. Bacteriophages were isolated using the double-layer agar plate method (Clokie, 2009). Briefly, approximately 100 g of offshore sedimentse was mixed with 300 mL of 2216E liquid culture and 50 mL of *Vibrio parahaemolyticus* and cultivated at 28°C, 180 rpm for 5 days. Samples (10 mL) were collected at 24, 72, and 120 h (Ding et al., 2020). After centrifugation, the supernatant was filtered with 0.22-μm membrane and diluted in sterile PBS. Then 100 μL of diluted supernatant was mixed with 100 μL of *Vibrio parahaemolyticus* by incubation at 28°C for 10 min. Finally, add 6 mL 2216E semisolid medium, pour it on the surface of hard agar plate and incubate overnight at 37°C. The plaque was purified 6 times until the plaque with the same size and shape was obtained to ensure the purity of the phage stock (Wang et al., 2019; Ding et al., 2020).

### Observing bacteriophage morphology using TEM

To identify the morphological characteristics, the morphology of bacteriophage PGA and PGB were characterized using the TEM. Briefly, the purified bacteriophage supernatants were added onto the surface of a copper grid and adsorbed at room temperature for 15 min. The bacteriophages were negatively stained with 2% phosphotungstic acid in darkness for 30 s and the morphological characteristics were observed under TEM.

### Bacteriophage whole genome sequencing and phylogenetic analysis

Genomic DNA of bacteriophage was extracted using a Genomic DNA Mini Kit. DNA concentration and purity were measured using NanoDrop One at the same time. ALFA-SEQ DNA Library Prep kit was used for Library-building operations. The library quality was assessed by the Qubit 4.0 Fluorometer and Qsep400 High-Throughput Nucleic Acid Protein Analysis system. Then prepared DNA samples were initially fragmented randomly to generate DNA fragments of desired lengths. The sticky ends resulting from the fragmentation were then repaired to create blunt ends. Subsequently, a specific adapter with a 3′ end containing a “T” base was ligated to the repaired DNA fragments by adding a base “A” at the 3′ end of the fragments. Finally, PCR amplification was performed to amplify the DNA fragments with the ligated adapters at both ends, completing the construction of the entire library. The constructed and qualified library was subjected to cluster preparation and sequencing on an Illumina Novaseq 6000 platform. To control the quality, Soapnuke was employed to eliminate low-quality sequencing data and duplicate data generated during PCR (Chen et al., 2018). Then BWA removed the host sequences from the reads (Li and Durbin, 2009). The trimmed reads were assembled by metaviralspade. In addition, ORFs of the bacteriophage genome were predicted by the GeneMarks online server and ORF Finder (Stalin and Srinivasan, 2017). In addition, the GeneMarks online server and ORF Finder were used to predict ORFs in the bacteriophage genome (Stalin and Srinivasan, 2017). Translated ORFs are annotated against NCBI’s non-redundant protein database by BLASTP algorithm (E value < 0.001). To predict the prophage and antibiotics resistance, we used the web tools VirulenceFinder (Joensen et al., 2014) and ResFinder server (Zankari et al., 2012). The two bacteriophage genomes were uploaded to the ViPTree for phylogenetic analysis (Nishimura et al., 2017). We compared the bacteriophages with other reported bacteriophages by progressive Mauve algorithm (Darling et al., 2004; Li et al., 2022).

### Phage ability and application analysis

In the prediction of bacteriophage hosts, BLASTn was used for preliminary analysis (Altschul et al., 1990), followed by the refinement of the analysis results using DeepHost (Ruohan et al., 2022). The dependent database is NCBI taxonomy database^1^ (Federhen, 2012). Graphage was used to predict the lytic activity of bacteriophages (Wang et al., 2022) with its database derived from TemPhD^2^ (Zhang X. et al., 2022; Zhang M. et al., 2022) and the NCBI RefSeq database^3^ (O’Leary et al., 2016). The genes conferring bacteriophage resistance against bacterial CRISPR systems were analyzed by MMseqs2 (Steinegger and Söding, 2017) and AcRanker (Eitzinger et al., 2020), and reference data was from Anti-CRISPRdb^4^ (Dong et al., 2018). MMseqs2 was also used to analyze the safety of bacteriophages (Steinegger and Söding, 2017). It involved searching the bacteriophage genome for antibiotic resistance genes and virulence genes in the CARD^5^ (McArthur et al., 2013) and VFDB^6^ (Chen et al., 2005) databases.

### Host range of bacteriophage

The host range of bacteriophage PGA and PGB on the *Vibrio parahaemolyticus* strains was determined by the double-layer agar plate method for 10 bacteria strains (Table 1; Pujato et al., 2017; Moodley et al., 2019; Feng et al., 2021). For this, 100 μL of *Vibrio parahaemolyticus* was added into 6 mL of 2216E semisolid medium, pour it on the surface of hard agar plate. After curing, 5 μL bacteriophage liquid was dripped onto the plate and incubated overnight at 37°C to allow cell lysis by the isolated bacteriophages, then their plaque formation is monitored. Check whether lysis has occurred, judging from the clarity of lysis: (−) no lysis; (+) weak lysis; (++) lysis; (+++) strong lysis (Liang et al., 2022).

### One-step growth curve

The one-step growth experiment was carried out as mentioned above, and some modifications were made (Hagens and Loessner, 2010). In brief, bacteriophage was mixed with *Vibrio parahaemolyticus* at MOI of 0.1 and incubated at 28°C for 20 min. After centrifugation at 8,000 rpm for 10 min to remove unabsorbed free bacteriophage, the mixture of bacteriophage and bacteria was washed with 2216E for three times. When they were harvested by centrifugation, the sediments of bacterial cells and bacteriophages were suspended with 200 mL of fresh 2216E, and incubated with shaking at 28°C at a speed of 150 rpm. The moment was defined as t = 0 min, and samples are collected at intervals of 10 or 30 min (0, 10, 20, 30, 40, 50, 60, 90, and 120 min) before the bacteriophage titer of each sample is determined by the double-layer method. The outbreak size was calculated as the ratio of the final count of bacteriophage particles released during the incubation period to the initial count of infected bacterial cells. One-step growth curves was drawn, and the incubation period, rising period and bacteriophage burst size of bacteriophage PGA and PGB were calculated as mentioned above (Pujato et al., 2015).

### Bacteriophage lytic activity against *Vibrio parahaemolyticus* in liquid culture

The antibacterial effect of bacteriophage PGA and PGB against *Vibrio parahaemolyticus* was detected in a 96-well plate (Liang et al., 2022). Briefly, bacteriophage was added into the *Vibrio parahaemolyticus* as MOI 0.01, 0.1, 1, and 10. Then 200 μL of the mixture was added into a 96-well plate. The blank group was 2216E liquid medium, and the control group was *Vibrio parahaemolyticus*. The 96-well plate was placed in the Microplate Reader (28°C, low speed) for culture. Starting from 0 min, the absorbance (OD_600_) was detected for each sample every 10 min.

### Isolation of anti-phage mutant strain

To obtain *Vibrio parahaemolyticus* mutants resistant to bacteriophage, following the method described (Yang et al., 2020) as described previously with some modifications. Briefly, 100 μL of bacteriophage was added into 10 mL of exponential phase *Vibrio parahaemolyticus* (OD_600_ = 0.1), the culture was cultivated on a shaker at 28°C with shaking for 3 days and the OD_600_ was monitored.

### Bacteriophage adsorption assays

Bacteriophage adsorption test was carried out by *Vibrio parahaemolyticus* (Le et al., 2014). Briefly, bacteriophage was added with an MOI of 0.01 and adsorbed at 28°C for 20 min. Then the bacteriophage and bacteria mixture were washed three times with PBS. Finally, the morphology of mixture was characterized using the transmission electron microscopy (Yang et al., 2020).

### Transcriptomics and analysis of differentially expressed genes

Genome Sequencing was performed by Magigene Biotechnology Co., Ltd. (Guangzhou, China). The expression level of transcripts were quantified by Salmon (Patro et al., 2017). DEGs were performed using the edgeR (Robinson et al., 2010). GO and KEGG enrichment analysis of DEGs were implemented by the clusterProfiler.

### Growth assays in Biolog GEN III MicroPlates

Wild strains and the anti-phage mutant strain of *Vibrio parahaemolyticus* were analyzed by Biolog GEN III MicroPlates. The growth assays of bacteriophages PGA and PGB was tested according to the method described by Williams et al. (2017).

### Statistical analysis

In order to calculate the average value and standard deviation of the numerical data of three independent experiments, the variance analysis method is used. The differences between treatments were analyzed by *t*-test.

### Nucleotide sequence accession number

The complete genome sequence of bacteriophage PGA and PGB have been deposited in the GenBank database under the accession number PP001175 and PP001176. Transcriptional sequencing of *Vibrio parahaemolyticus* have been deposited in the BioProject database under the accession number PRJNA1055304.

## Results

### Isolation and authenticate of bacteriophage

#### vB_VpaS_PGA and vB_VpaS_PGB morphology

**Figure 1:**
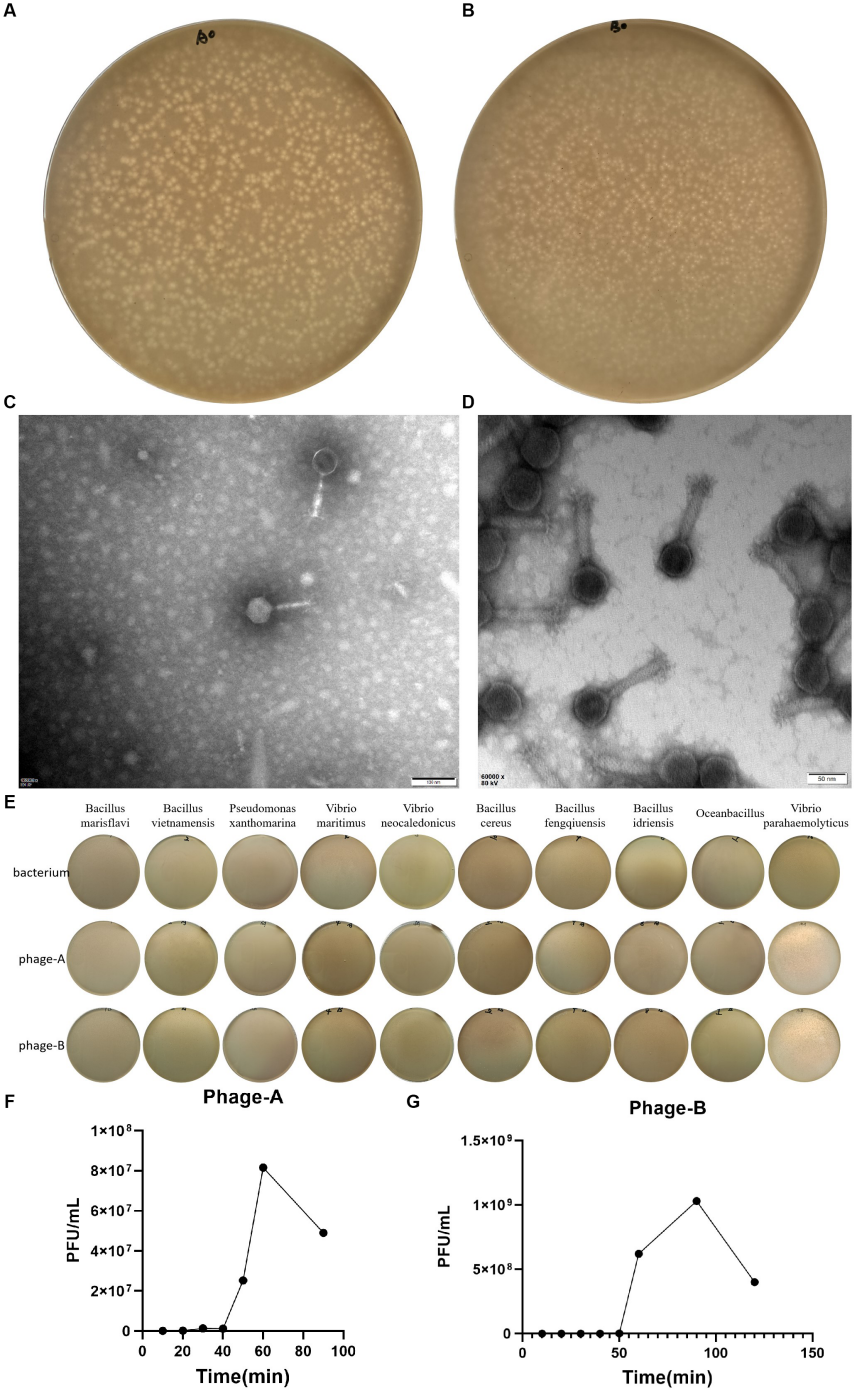
Characterization of Bacteriophage. **(A)** Plaques of bacteriophage vB_VpaS_PGA. **(B)** Plaques of bacteriophage vB_VpaS_PGB. **(C)** Transmission electron microscopy of bacteriophage vB_VpaS_PGA. **(D)** Transmission electron microscopy of bacteriophage vB_VpaS_PGB. **(E)** Host range of bacteriophage vB_VpaS_PGA and vB_VpaS_PGB. **(F)** One-step growth curve of PGA. **(G)** One-step growth curve of PGB.

We induced and isolated two bacteriophage that could target the *Vibrio parahaemolyticus*, obtained from the offshore sedimentse of a large-scale aquaculture areas. Bacteriophage PGA and PGB formed clear plaques (about 1.6 mm in diameter of PGA and about 1 mm in diameter of PGB) on the double-layered agar plate (Figures 1A,B), indicating that the bacteriophage was lytic. After staining with 2% PTA negatively, the TEM image showed that PGA (Figure 1C) and PGB (Figure 1D) consist of an icosahedral head and a long non-contractile tail. The measurements of length of head, diameter of head, and length of contractile tails of PGA were 66.5 ± 2, 61 ± 1, and 100 ± 5 nm while those of PGB were 72.5 ± 2, 70.5 ± 1, and 136 ± 5 nm. By the International Virus Classification and Nomenclature, bacteriophages PGA and PGB belong to the family *Myoviridae*, order *Caudovirales* (Lefkowitz et al., 2018). Bacteriophages PGA and PGB belonged to the order *Myoviridae* and then were named as vB_VpaS_PGA (bacteriophage PGA) and vB_VpaS_PGB (bacteriophage PGB).

#### Host range

To determine the host range of bacteriophages PGA and PGB, the double-layer agar plate method was used for 10 bacteria strains. As the results (Figure 1E; Table 1), bacteriophage PGA and PGB can only lyse *Vibrio parahaemolyticus*. These results indicated that these phages had a narrow host range which can specifically target *Vibrio parahaemolyticus* strains, suggesting the potential of the bacteriophage PGA and PGB to be a candidate for bacteriophage therapy (Liang et al., 2022).

#### Biological characteristics of vB_VpaS_PGA and vB_VpaS_PGB

The life cycle of bacteriophage, including the latent period, explosive phase, and plateau phase, was quantified using a one-step growth curve. As shown in Figure 1F, PGA was characterized by a short incubation period of 20 min, an outbreak period of 60 min and a burst size of 88.4 PFUs/infected cell, indicating that phages grew efficiently and rapidly after adsorption on the host surface. The latent period and burst period of phage PGB were 50 and 90 min, and the burst size was about 222.0 PFUs/infected cell (Figure 1G). Taking these together, bacteriophages PGA and PGB have a good lysis effect to *Vibrio parahaemolyticus* (Liang et al., 2022).

#### Phage genome analysis

**Figure 2:**
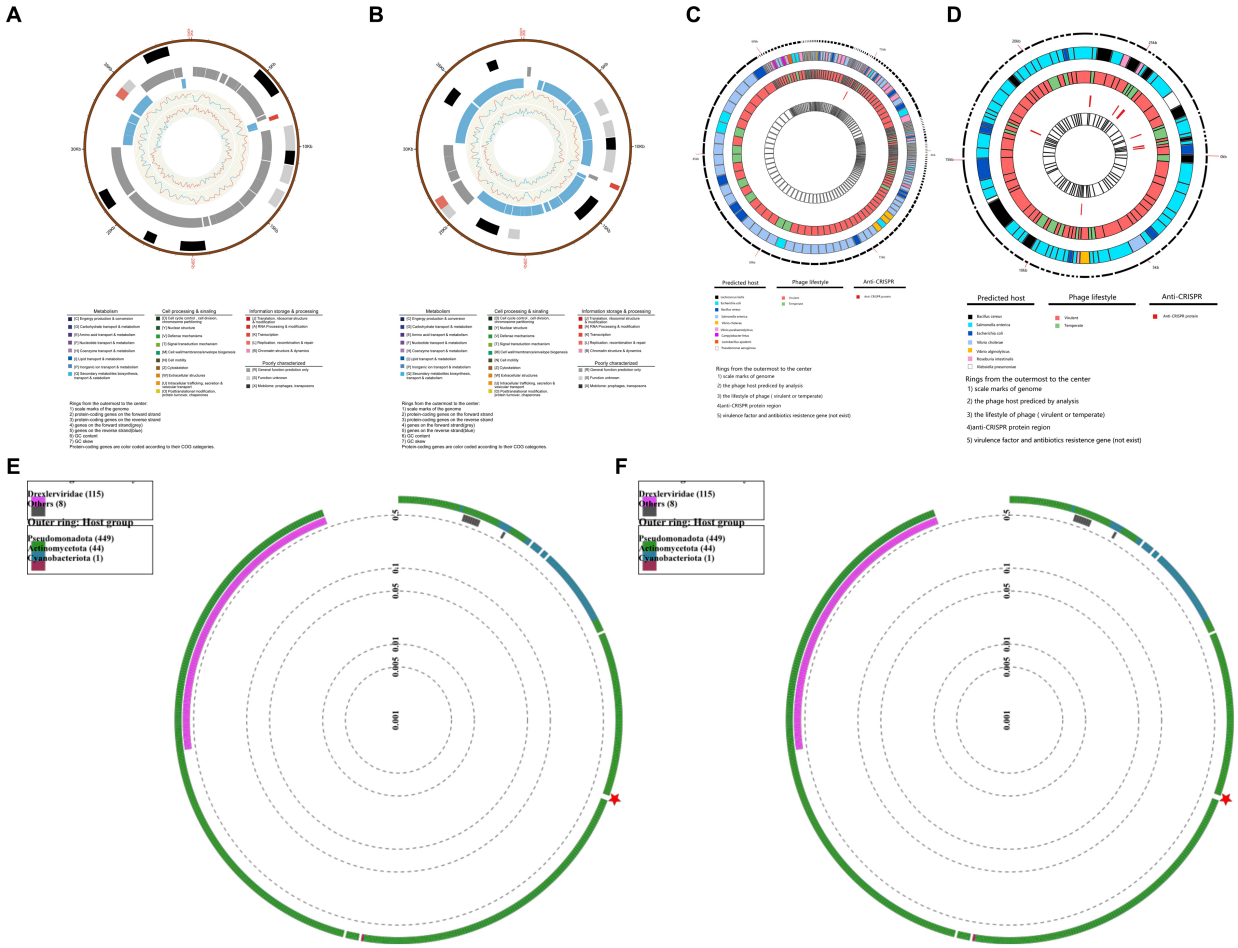
Genomic characterization of bacteriophages. **(A)** Genomic characterization of vB_VpaS_PGA. Rings from the outermost to the center: (1) scale marks of the genome; (2) protein-coding genes on the forward strand; (3) protein-coding genes on the reverse strand; (4) genes on the forward strand (gray); (5) genes on the reverse strand (blue); (6) GC content; (7) GC skewProtein-coding genes are color coded according to their COG categories. **(B)** Genomic characterization of vB_VpaS_PGB. Rings from the outermost to the center: (1) scale marks of the genome; (2) protein-coding genes on the forward strand; (3) protein-coding genes on the reverse strand; (4) genes on the forward strand (gray); (5) genes on the reverse strand (blue); (6) GC content; (7) GC skewProtein-coding genes are color coded according to their COG categories. **(C)** Security and efficacy of vB_VpaS_PGA. Rings from the outermost to the center: (1) scale marks of genome; (2) the phage host predicted by analysis; (3) the lifestyle of phage (virulent or temperate); (4)anti-CRISPR protein region; (5) virulence factor and antibiotics resistance gene (not exist). **(D)** Security and efficacy of vB_VpaS_PGA. Rings from the outermost to the center: (1) scale marks of genome; (2) the phage host predicted by analysis; (3) the lifestyle of phage (virulent or temperate); (4)anti-CRISPR protein region; (5) virulence factor and antibiotics resistance gene (not exist). **(E)** Phylogenetic analysis of vB_VpaS_PGA (circular). The phylogenetic tree was constructed using the Maximum Likelihood method with 1,000 bootstrap replicates. Amino acid sequences of the terminase large subunit. **(F)** Phylogenetic analysis of vB_VpaS_PGB (circular). The phylogenetic tree was constructed using the Maximum Likelihood method with 1,000 bootstrap replicates. Amino acid sequences of the terminase large subunit.

In order to understand the Genetic characteristics of bacteriophages, the genome of bacteriophage PGA and PGB were sequenced and analyzed (Yang et al., 2020). Genomic characterization of the two bacteriophages indicated (Figures 2A,B) that the genomes of PGA and PGB are circular double-stranded DNA (of 40.27 and 40.27 bp, respectively). The GC content of bacteriophage PGA (42.26%) was similar to PGB (42.28%; Li et al., 2022). In addition, the results (Table 2) show that although 63 and 62 ORFs were identified in PGA and PGB respectively, only 28.57% and 27.42% of the ORFs were assigned to specific functions (DNA replication, DNA metabolism, DNA packaging, and structure formation).

**Table 2:** Basic genome characteristics of PGA, PGB.

Characteristic	PGA	PGB
Genome length (bp)	40.27	40.27
No. of GC content (%)	42.26	42.28
ORFs	63	62
Functionally annotated ORF	18	17
Virulence gene	0	0
Antimicrobial resistance gene	0	0

**Table 3:** The names of carbon sources in each well.

A1 negative control	A2 Dextrin	A3 D-Maltose	A4 D-Trehalose	A5 D-Cellobiose	A6 Gentiobiose	A7 Sucrose	A8 D-Turanose	A9 Stachyose	A10 positive control	A11 pH 6	A12 pH 5
B1 D-Raffinose	B2 α-D-Lactose	B3 D-Melibiose	B4 β-Methyl-D-Glucoside	B5 D-Salicin	B6 N-Acetyl-D-Glucosamine	B7 N-Acetyl-β-DMannosamine	B8 N-Acetyl-D-Galactosamine	B9 N-AcetylNeuraminic Acid	B10 1% NaCl	B11 4% NaCl	B12 8% NaCl
C1 α-D-Glucose	C2 D-Mannose	C3 D-Fructose	C4 D-Galactose	C5 3-Methyl Glucose	C6 D-Fucose	C7 L-Fucose	C8 L-Rhamnose	C9 Inosine	C10 1% Sodium Lactate	C11 Fusidic Acid	C12 D-Serine
D1 D-Sorbitol	D2 D-Mannitol	D3 D-Arabitol	D4 myo-Inositol	D5 Glycerol	D6 D-Glucose-6-PO4	D7 D-Fructose-6-PO4	D8 D-Aspartic Acid	D9 D-Serine	D10 Troleandomycin	D11 Rifamycin SV	D12 Minocycline
E1 Gelatin	E2 Glycyl-L-Proline	E3 L-Alanine	E4 L-Arginine	E5 L-Aspartic Acid	E6 L-Glutamic Acid	E7 L-Histidine	E8 L-Pyroglutamic Acid	E9 L-Serine	E10 Lincomycin	E11 Guanidine HCl	E12 Niaproof 4
F1 Pectin	F2 D-Galacturonic Acid	F3 L-Galactonic Acid Lactone	F4 D-Gluconic Acid	F5 D-Glucuronic Acid	F6 Glucuronamide	F7 Mucic Acid	F8 Quinic Acid	F9 D-Saccharic Acid	F10 Vancomycin	F11 Tetrazolium Violet	F12 Tetrazolium Blue
G1 p-Hydroxy-PhenylaceticAcid	G2 Methyl Pyruvate	G3 D-Lactic Acid Methyl Ester	G4 L-Lactic Acid	G5 Citric Acid	G6 α-Keto-Glutaric Acid	G7 D-Malic Acid	G8 L-Malic Acid	G9 Bromo-Succinic Acid	G10 Nalidixic Acid	G11 Lithium Chloride	G12 Potassium Tellurite
H1 Tween 40	H2 γ-Amino-Butryric Acid	H3 α-Hydroxy-Butyric Acid	H4 β-Hydroxy-D,LButyric Acid	H5 α-Keto-Butyric Acid	H6 Acetoacetic Acid	H7 Propionic Acid	H8 Acetic Acid	H9 Formic Acid	H10 Aztreonam	H11 Sodium Butyrate	H12 Sodium Bromate

The potential applications of bacteriophages were analyzed via bioinformatics tools, and the results were visualized using TBtools-II in Figures 2C,D (Chen et al., 2023). The genome structure and ORFs of PGA and PGB were determined from previous whole genome sequencing analysis. BLASTn and DeepHost were utilized to analyze potential hosts for the bacteriophage and map them to their corresponding positions on the genome. From the analysis of bacteriophage lifestyle, it is evident that both PGA and PGB demonstrate pronounced bacteriolytic activity against their respective hosts. AcRanker and MMseq2 revealed a higher abundance of anti-CRISPR system genes in PGB compared to PGA, which exhibited a lower presence of such genes. Based on the alignment and comparison results using MMseqs2, the presence of virulence genes and antibiotic resistance genes was not detected in both PGA and PGB (as shown in the innermost circles in Figures 2C,D). PGA and PGB meet the prerequisites (non-virulence genes, antimicrobial resistance genes or lysogenic genes) of bacteriophage therapy candidates, so they can be used as specific lytic bacteriophages (Bardina et al., 2016; Li et al., 2021). In summary, the analyses have demonstrated the strong lytic activity of PGA and PGB toward their host, as well as the absence of virulence genes and drug resistance genes, thus ensuring the genetic safety of these phages for applications. The bioinformatics-based predictions and validations contribute to assessing the security and efficacy of utilizing these two phage strains for therapeutic interventions.

We found that the phylogenetic analysis (Figures 2E,F) revealed that PGA and PGB had the closest relationship withVibrio bacteriophage vB_VpP_BT-1011 (NC_070774). The homology of PGA and PGB with vB_VpP_BT-1011 was 75.86% and 76.35% respectively, only two Vibrio bacteriophages appear in the corresponding phylogenetic tree and it shows that they are new species of this genus (Li et al., 2022).

### Anti-phage mutant strain showed clear resistance to bacteriophage PGA/PGB

**Figure 3:**
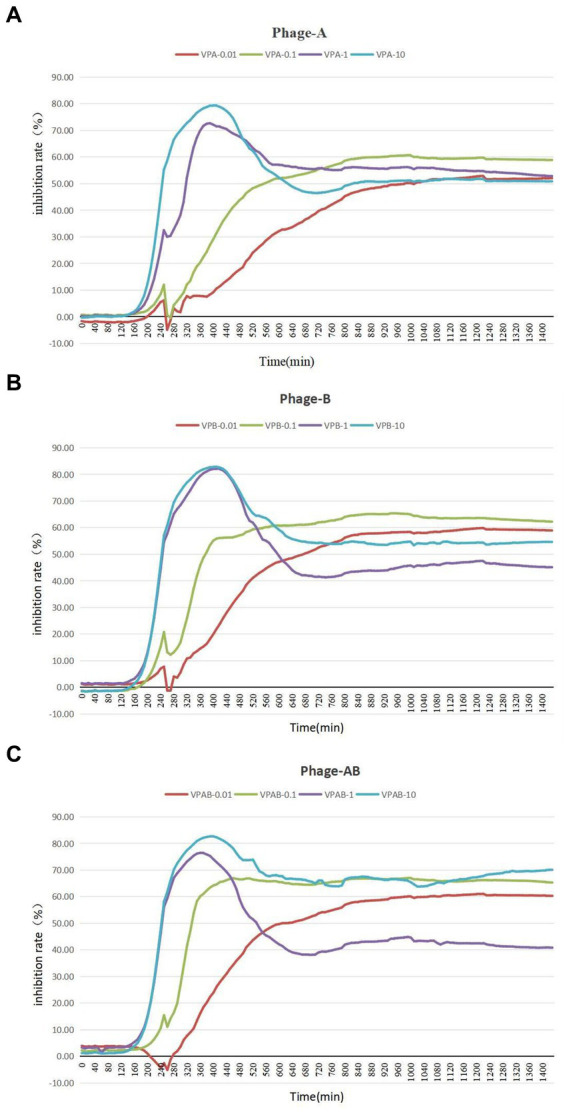
Lysis of *Vibrio parahaemolyticus* strains by vibriophage vB_VpaS_PGA and vB_VpaS_PGB. vB_VpaS_PGA and vB_VpaS_PGB infected different *Vibrio parahaemolyticus* strains of infection (MOI), and the densities of the cultures were determined by measuring absorbance at 600 nm. **(A)** Bacteriophage vB_VpaS_PGB. **(B)** Bacteriophage vB_VpaS_PGB. **(C)** Bacteriophage vB_VpaS_PGA and bacteriophage vB_VpaS_PGB.

**Figure 4:**
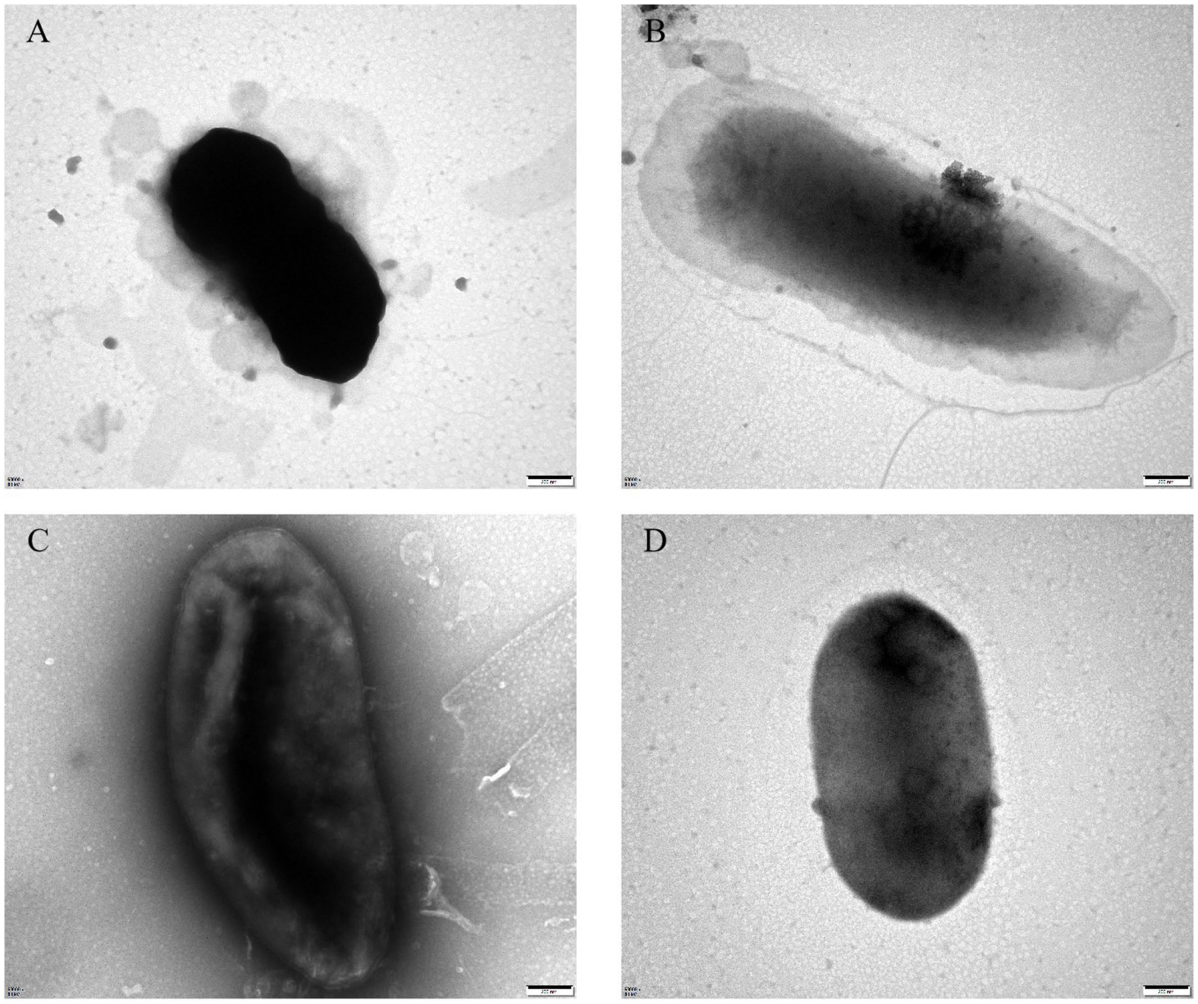
Transmission electron microscopy of bacteriophage adsorbing *Vibrio parahaemolyticus.* **(A)** Bacteriophage vB_VpaS_PGA adsorption of the wild-type strain. **(B)** Bacteriophage vB_VpaS_PGA adsorption of the anti-phage mutant strain. **(C)** Bacteriophage vB_VpaS_PGB adsorption of the wild-type strain. **(D)** Bacteriophage vB_VpaS_PGB adsorption of the anti-phage mutant strain.

To assess the growth-inhibitory effect of bacteriophages PGA and PGB on *Vibrio parahaemolyticus*, the growth curve was measure by bacteriophage PGA/PGB. As the results (Figures 3A,B), when the MOI was 1 and 10 respectively, the growth of *Vibrio parahaemolyticus* was obviously inhibited within 8 h, and then increased exponentially. Meanwhile *Vibrio parahaemolyticus* regrew after 4 h at MOI of 0.01 and 0.1. This means that *Vibrio parahaemolyticus* regrows after adding single bacteriophage. Then we determine the growth-inhibitory effect of the mixed bacteriophage on *Vibrio parahaemolyticus*. The results (Figure 3C) again revealed spontaneous mutation of *Vibrio parahaemolyticus* occurred during co-culture. Based on the bacteriophage co-culture assay, we hypothesized that spontaneous mutation of *Vibrio parahaemolyticus* may lead to the inhibition of bacteriophage adsorption. In order to prove this hypothesis, the anti-phage mutant strain were obtained after 17 rounds of co-culture of *Vibrio parahaemolyticus* and mixed bacteriophage. The anti-phage mutant strain was designated as the anti-phage mutant strain VP-17. Then whether the bacteriophage was adsorbed on *Vibrio parahaemolyticus* was observed by transmission electron microscope. The results (Figures 4A–D) confirmed that spontaneous mutation of *Vibrio parahaemolyticus* result in the inhibition of bacteriophage adsorption.

### Genomic analysis of differences between the wild-type strain and the anti-phage mutant strain

**Figure 5:**
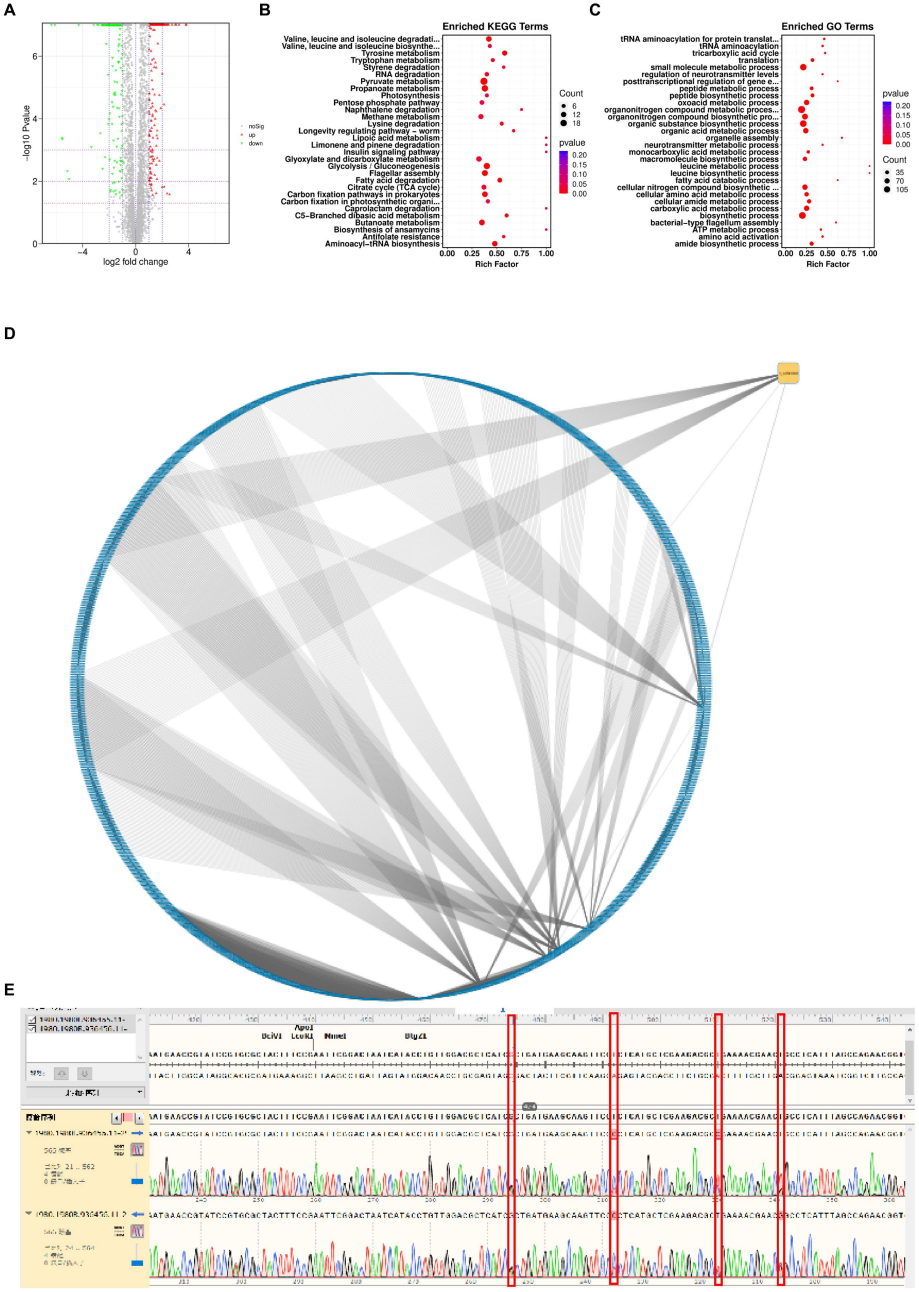
Genetic changes from the wild-type strain to the anti-phage mutant strain. **(A)** Differential gene volcanic map. **(B)** KEGG function enrichment analysis statistical chart. **(C)** GO function enrichment analysis statistical chart. **(D)** Network diagram of interaction relationship between genes with mutation sites and differentially expressed genes. Blue is the differentially expressed gene, and yellow is the gene with mutation site. From an interactive point of view, there is variation information. Gene 1_orf 01980 interacts with 90 different genes. **(E)** SNP comparison.

To determine the genomic changes associated with bacteriophage adsorption, multigroup sequencing was carried out on spontaneous the anti-phage mutant strain (VP-17) and the wild-type strain (MCCC 1A16298; Martin et al., 2018). We found 25 SNP differences by comparing the core genomes of the wild-type strains and the anti-phage mutant strain (Supplementary Table S2). Since an spontaneous mutation was observed in the co-culture by *Vibrio parahaemolyticus* (Figure 3C), it is expected that the mutant gene of bacteriophage resistance can be identified by differential RNA-seq. Specifically, the results of differential expression analysis showed that compared with wild-type strains, 316 genes were up-regulated and 466 genes were down-regulated in phage-resistant mutant (Figure 5A). On the whole, genes related to pyruvate metabolism, propanoate metabolism, glycolysis/gluconeogenesis and flagellar assembly (Figure 5B) were found to be more differentially expressed when spontaneous mutation of *Vibrio parahaemolyticus* occurred during co-culture. The function of these genes are closely related to the survival of bacteria and their adaptation to stimuli (Kim and McCarter, 2000), and the results of GO enrichment analysis also confirmed this result (Figure 5C). The analysis of the interaction between the gene where the mutation site is located and the differentially expressed gene shows that the gene 1_orf 01980 where the mutation site is located has an interaction relationship with 90 differential genes (Figure 5D). The gene 1_orf 01980 prediction is related to flaG gene, flaG gene is a flagella gene, which has chemotaxis and motility (Ahmmed et al., 2019). In order to verify the accuracy of the mutation site, we used the first generation sequencing to sequence SNPs. The result (Figure 5E) showed that the anti-phage mutant strain did exist SNP mutation sites.

### Trade-off of bacteriophage resistance and growth competitiveness

**Figure 6:**
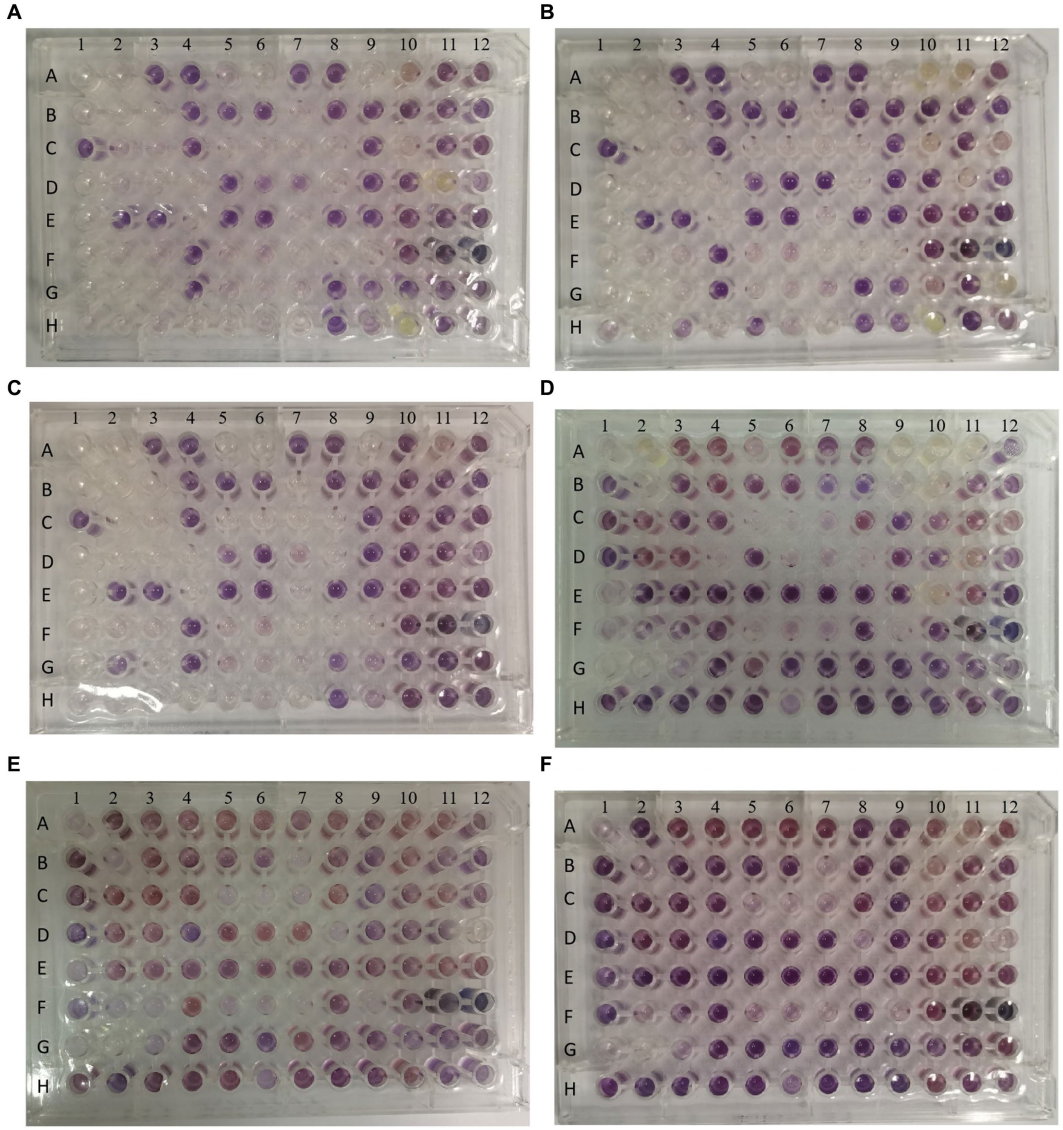
Picture of one representative set of Biolog plates after 24 h of incubation. From left to right: Wells A1 and A9 of each plate are the negative and positive controls. Chemical indicator assays are in columns 10–12. The names of carbon sources in each well are provided in Table 3. **(A–C)** The three independent biological replicates of the wild-type strain. **(D–F)** The three independent biological replicates of the anti-phage mutant strain.

**Figure 7:**
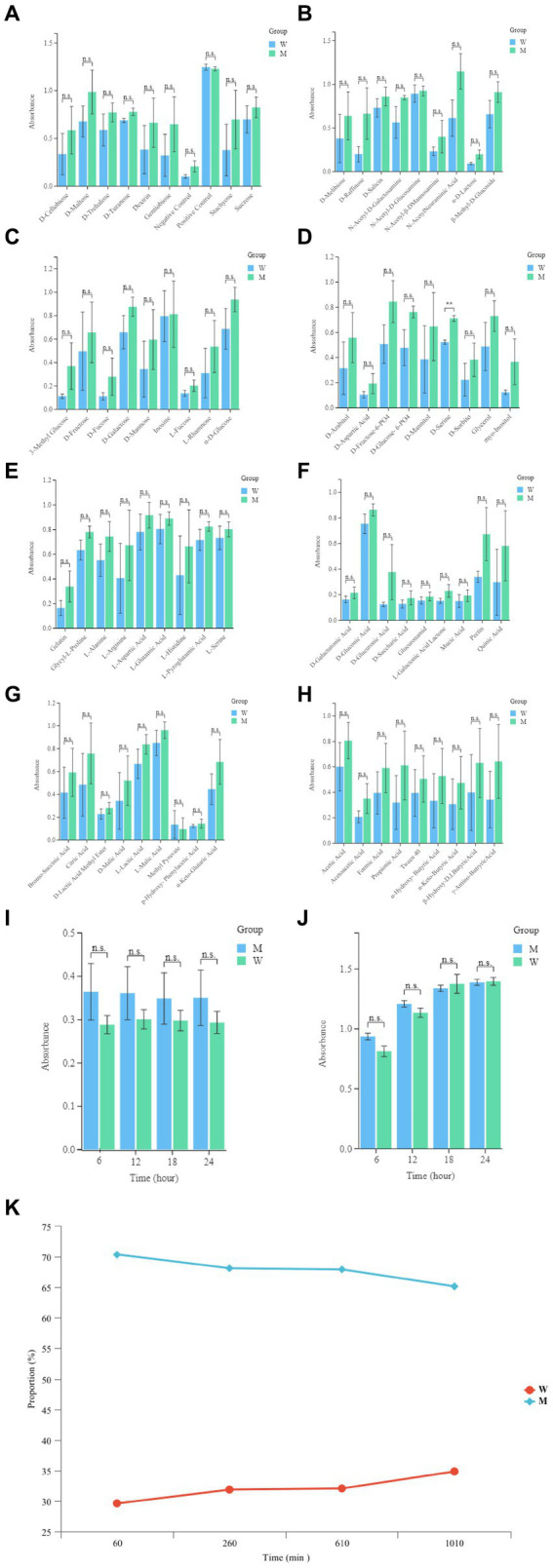
Comparative analysis of growth of the wild-type strain and the anti-phage mutant strain. **(A)** The absorbance readings of the negative control wells, the raw spectrophotometric triplicate data collected from each experimental well at 6, 12, 18, and 24 h was directly analyzed. **(B)** The absorbance readings of the positive control well, the raw spectrophotometric triplicate data collected from each experimental well at 6, 12, 18 and 24 h was directly analyzed. **(C–J)** Each panel corresponds to a row in the Biolog plate, respectively. The raw data absorbance values for triplicate samples from the 24 h time point were averaged. The names of the carbon sources in wells 1–9 within each row are provided on the x-axis. Each set of data represent the wild-type strain and the anti-phage mutant strain from left to right, respectively. Error bars were calculated using the standard error across the three independent biological replicates. **(K)** The growth competitiveness of the wild strains and the anti-phage mutant strain, Compare the proportions of the wild strains and the anti-phage mutant strain at different time points (60, 260, 610, and 1,010 min).

In order to evaluate the metabolic capacity of the wild-type strain and the anti-phage mutant strain, Biolog GEN III MicroPlates were used to analyze this capacity. After 24 h of incubation, MCCC 1A16298 and VP-17 strains had differences in carbon source utilization on the Biolog GEN III MicroPlate (Figures 6A–F) by naked eye observation, it shows that there are differences between the two kinds of bacteria in their ability to use carbon sources. Statistical analysis via a *t*-test (Figures 7A,B) showing that every negative control of MCCC 1A16298 and VP-17 did not increase significantly, and no statistically significant differences in the absorbance of the positive control, it shows that the growth rate in complete culture medium was similar. At these four time points (6, 12, 18, and 24 h), the data trend of all carbon source were consistent. Therefore, we use data of 24 h time point for statistical analysis. Of particular surprise, the results (Figures 7C–J) showed that there were no statistically significant differences between VP-17 and MCCC 1A16298. Then the growth competitiveness of the wild strains and the anti-phage mutant strain was also tested, the two kinds of bacteria were mixed in a certain proportion, and samples were taken and sequenced at different time points (60, 260, 610 and 1,010 min). The results (Figure 7K) show that with the passage of time, the proportion of the anti-phage mutant strain is decreasing, while the proportion of the wild-type strains is increasing. In other words, the growth competitiveness of the wild-type strain is stronger than that of the anti-phage mutant strain. The above data demonstrated that the anti-phage mutant strain obtained bacteriophage resistance at the cost of growth competitiveness.

## Discussion

With the continuous deterioration of antibiotic resistance and the continuous growth of drug-resistant pathogens, bacteriophage therapy is favored (Carlton, 1999; Lu and Collins, 2007, 2009). As early as 1919, bacteriophage therapy treated chickens infected with *Salmonella gallinarum* (Sulakvelidze et al., 2001). In the past 20 years, many researchers have studied the identification and clinical application of bacteriophage (Dedrick et al., 2019; Jault et al., 2019). At present, most of the research on bacteriophage is based on sequencing to predict its function. For bacteriophage therapy, it is necessary to solve the development of bacteriophage resistance to the host. However, the mechanism of bacteriophage resistance is rarely reported, we lack detailed knowledge about their ecological and evolutionary interactions (Kortright et al., 2019; Markwitz et al., 2021; North and Brown, 2021; Tang et al., 2022).

The interactions between bacteriophages and the anti-phage mutants are complex. To gain insight into such relationships, we screened a collection of 2 vibriophages (PGA and PGB) from the offshore sedimentse samples. It is evident that both PGA and PGB demonstrate pronounced bacteriolytic activity against their respective hosts. Most importantly, no virulence gene was identified showing the safety of vibriophages PGA and PGB in biocontrol applications. To resist bacteriophage infection, host genes sometimes evolve mutations and have a significant impact on their own metabolic activities. In this study, it was found that the anti-phage mutants can not be adsorbed by bacteriophage because of the mutation of flaG gene (slight homology to N terminus of multiple flagellins) mutated (Figure 5E; Sar et al., 1990; Ahmmed et al., 2019). It is reported that through modifying cell surface to prevent bacteriophage attachment and entry, bacteriophage adsorption is blocked, resulting in bacteriophage resistance (Destoumieux-Garzón et al., 2005; Samson et al., 2013; Hill et al., 2018). Specifically, the mutation of cell surface receptors (fimbriae, flagella, outer membrane proteins, and lipopolysaccharide) is very important to inhibit the binding of bacteriophage to the host bacteria (Qimron et al., 2006; Westra et al., 2015; Houte et al., 2016; Shen and Loessner, 2021).

In addition, the growth competitiveness of wild strains and the anti-phage mutants was also tested, it was found that the growth competitiveness of the anti-phage mutant strain is lower than the wild-type strain. It is reported that under the pressure of bacteriophage selection, bacteria evolve bacteriophage resistance through bacterial defense systems (Bikard and Marraffini, 2012; Samson et al., 2013). Anti-phage mutant strain may make trade-offs with bacterial growth rate, toxicity, drug resistance, utilization of carbon source and formation of biofilm (Mangalea and Duerkop, 2020; Castledine et al., 2022), which is consistent with our results. In the natural environment, the bacterial community are diverse and the bacterial population are dynamic. Bacteria compete for resources and follow the principle of competition and exclusion, and those bacteria with slow growth rates will be eliminated. Although the anti-phage mutants acquired resistance to phage, their growth competitiveness also declined. Under the condition of competitive exclusion principle, the mutant strain is not competitive and may be eliminated, which opens up a new idea for phage therapy to produce drug resistance. In the natural environment, few living things exist in a constant environment, and trade-offs will be different in different environments. This study also provides an insight into the co-evolution of trade-offs from the perspective of the evolution of microbial laboratory.

Overall, this characterization and analysis provides a theoretical basis for the application of bacteriophage therapy. Bacteriophage treatment can not only enhance the resistance to bacteriophage, but also enhance the sensitivity to antibiotics (German and Misra, 2001; Chan et al., 2016). The positive effect of bacteriophage resistance can improve the sensitivity of antibiotics, which can make up for the deficiency of bacteriophage therapy (Wang et al., 2021). This provides an idea for treatment and can be used together with other reagents (such as antibiotics and lyases). This study provides a basis for further study on the relationship between related bacteriophages of *Vibrio parahaemolyticus*. In the future, it is necessary to further study the diversity of bacteriophage and its biological control mechanism against bacteria, especially pathogens, and more work is needed to understand the complexity of pleiotropic interaction between various bacteriophages and bacteria. However, this research has its limitations. The environment of this experiment is too single simple, the medium used to isolate and cultivate microorganisms in the laboratory has a strong selection function. This method can not fully reflect the ecological function of marine microorganisms (Bacteria and bacteriophages), for the dynamic changes during phage therapy, it is very important to better understand the community evolution and ecological variability.

## Data availability statement

The authors acknowledge that the data presented in this study must be deposited and made publicly available in an acceptable repository, prior to publication. Frontiers cannot accept a manuscript that does not adhere to our open data policies.

## Author contributions

XZ: Data curation, Methodology, Writing – original draft. SL: Writing – original draft, Resources. GG: Resources, Writing – review & editing, Software. YH: Writing – review & editing, Conceptualization. YS: Writing – review & editing, Supervision, Funding acquisition. JD: Writing - review & editing, Data curation.

## Footnotes

^1^ https://www.ncbi.nlm.nih.gov/taxonomy

^2^ https://phage.deepomics.org/

^3^ https://www.ncbi.nlm.nih.gov/refseq/

^4^ http://guolab.whu.edu.cn/anti-CRISPRdb/

^5^ https://card.mcmaster.ca/

^6^ http://www.mgc.ac.cn/VFs/main.htm

## References

[ref1] Aarestrup F. M. Wegener H. C. (1999). The effects of antibiotic usage in food animals on the development of antimicrobial resistance of importance for humans in campylobacter and *Escherichia coli*. Microbes Infect. 1, 639–644. doi: 10.1016/s1286-4579(99)80064-1, PMID: 10611741

[ref2] Ahmmed S. Khan M. A. A. K. Eshik M. M. E. Punom N. J. Islam A. B. M. M. K. Rahman M. S. (2019). Genomic and evolutionary features of two AHPND positive *Vibrio parahaemolyticus* strains isolated from shrimp (*Penaeus monodon*) of south-West Bangladesh. BMC Microbiol. 19, 1–14. doi: 10.21203/rs.2.14490/v2 31796006 PMC6889531

[ref3] Alagappan K. Karuppiah V. Deivasigamani B. (2016). Protective effect of phages on experimental *Vibrio parahaemolyticus* infection and immune response in shrimp (Fabricius, 1798). Aquaculture 453, 86–92. doi: 10.1016/j.aquaculture.2015.11.037

[ref4] Altschul S. F. Gish W. Miller W. Myers E. W. Lipman D. J. (1990). Basic local alignment search tool[J]. J. Mol. Biol. 215, 403–410. doi: 10.1016/S0022-2836(05)80360-2 2231712

[ref5] Bardina C. Colom J. Spricigo D. A. Otero J. Sánchez-Osuna M. Cortés P.. (2016). Genomics of three new bacteriophages useful in the biocontrol of salmonella. Front. Microbiol. 7:545. doi: 10.3389/fmicb.2016.00545, PMID: 27148229 PMC4837284

[ref6] Bikard D. Marraffini L. A. (2012). Innate and adaptive immunity in bacteria: mechanisms of programmed genetic variation to fight bacteriophages. Curr. Opin. Immunol. 24, 15–20. doi: 10.1016/j.coi.2011.10.005, PMID: 22079134

[ref7] Carlton R. M. (1999). Phage therapy: past history and future prospects. Arch. Immunol. Ther. Exp. (Warsz.) 47, 267–274. PMID: 10604231

[ref8] Castledine M. Padfield D. Sierocinski P. Pascual J. S. Hughes A. Mäkinen L.. (2022). Parallel evolution of *Pseudomonas aeruginosa* phage resistance and virulence loss in response to phage treatment in vivo and in vitro. eLife 11:e73679. doi: 10.7554/eLife.73679, PMID: 35188102 PMC8912922

[ref9] Chan B. K. Sistrom M. Wertz J. E. Kortright K. E. Narayan D. Turner P. E. (2016). Phage selection restores antibiotic sensitivity in MDR *Pseudomonas aeruginosa*. Sci. Rep. 6:26717. doi: 10.1038/srep26717, PMID: 27225966 PMC4880932

[ref10] Chan B. K. Turner P. E. Kim S. Mojibian H. R. Elefteriades J. A. Narayan D. (2018). Phage treatment of an aortic graft infected with *Pseudomonas aeruginosa*. Evolut Med Public Health 2018, 60–66. doi: 10.1093/emph/eoy005, PMID: 29588855 PMC5842392

[ref11] Chen Y. Chen Y. Shi C. Huang Z. Zhang Y. Li S.. (2018). SOAPnuke: a MapReduce acceleration-supported software for integrated quality control and preprocessing of high-throughput sequencing data. Gigascience. 7, 1–6. doi: 10.1093/gigascience/gix120, PMID: 29220494 PMC5788068

[ref12] Chen C. Wu Y. Li J. Wang X. Zeng Z. Xu J.. (2023). TBtools-II: a "one for all, all for one" bioinformatics platform for biological big-data mining. Mol. Plant 16, 1733–1742. doi: 10.1016/j.molp.2023.09.010, PMID: 37740491

[ref13] Chen L. Yang J. Yu J. Yao Z. Sun L. Shen Y.. (2005). VFDB: a reference database for bacterial virulence factors. Nucleic Acids Res. 33, D325–D328. doi: 10.1093/nar/gki008, PMID: 15608208 PMC539962

[ref14] Clokie R. J. (2009). Kropinski Bacteriophages: methods and protocols. Methods Mol. Biol. 501:307. doi: 10.1007/978-1-60327-164-6

[ref15] Darling A. C. Mau B. Blattner F. R. Perna N. T. (2004). Mauve: multiple alignment of conserved genomic sequence with rearrangements. Genome Res. 14, 1394–1403. doi: 10.1101/gr.2289704, PMID: 15231754 PMC442156

[ref16] Dedrick R. M. Guerrero-Bustamante C. A. Garlena R. A. Russell D. A. Ford K. Harris K.. (2019). Engineered bacteriophages for treatment of a patient with a disseminated drug-resistant *Mycobacterium abscessus*. Nat. Med. 25, 730–733. doi: 10.1038/s41591-019-0437-z, PMID: 31068712 PMC6557439

[ref17] Destoumieux-Garzón D. Duquesne S. Peduzzi J. Goulard C. Desmadril M. Letellier L.. (2005). The iron–siderophore transporter FhuA is the receptor for the antimicrobial peptide microcin J25: role of the microcin Val11–Pro16 β-hairpin region in the recognition mechanism. Biochem. J. 389, 869–876. doi: 10.1042/BJ20042107, PMID: 15862112 PMC1180738

[ref9001] Ding T. Sun H. Pan Q. Zhao F. Zhang Z. Ren H. (2020). Isolation and characterization of Vibrio parahaemolyticus bacteriophage vB_VpaS_PG07. Virus Res. 286:198080. doi: 10.1016/j.virusres.2020.198080 32615132

[ref18] Dong C. Hao G. F. Hua H. L. Liu S. Labena A. A. Chai G.. (2018). Anti-CRISPRdb: a comprehensive online resource for anti-CRISPR proteins. Nucleic Acids Res. 46, D393–D398. doi: 10.1093/nar/gkx835, PMID: 29036676 PMC5753274

[ref19] Eitzinger S. Asif A. Watters K. E. Iavarone A. T. Knott G. J. Doudna J. A.. (2020). Machine learning predicts new anti-CRISPR proteins. Nucleic Acids Res. 48, 4698–4708. doi: 10.1093/nar/gkaa219, PMID: 32286628 PMC7229843

[ref20] Elmahdi S. DaSilva L. V. Parveen S. (2016). Antibiotic resistance of Vibrio parahaemolyticus and *Vibrio vulnificus* in various countries: a review. Food Microbiol. 57, 128–134. doi: 10.1016/j.fm.2016.02.008, PMID: 27052711

[ref21] Federhen S. (2012). The NCBI taxonomy database. Nucleic Acids Res. 40, D136–D143. doi: 10.1093/nar/gkr1178, PMID: 22139910 PMC3245000

[ref22] Feng T. Leptihn S. Dong K. Loh B. Zhang Y. Stefan M. I.. (2021). JD419, a *Staphylococcus aureus* phage with a unique morphology and broad host range. Front. Microbiol. 12:602902. doi: 10.3389/fmicb.2021.602902, PMID: 33967969 PMC8100676

[ref23] German G. J. Misra R. (2001). The TolC protein of *Escherichia coli* serves as a cell-surface receptor for the newly characterized TLS bacteriophage. J. Mol. Biol. 308, 579–585. doi: 10.1006/jmbi.2001.4578, PMID: 11350161

[ref24] Hagens S. Loessner J. M. (2010). Bacteriophage for biocontrol of foodborne pathogens: calculations and considerations. Curr. Pharm. Biotechnol. 11, 58–68. doi: 10.2174/138920110790725429, PMID: 20214608

[ref25] Hill C. Mills S. Ross R. P. (2018). Phages & antibiotic resistance: are the most abundant entities on earth ready for a comeback? Future Microbiol. 13, 711–726. doi: 10.2217/fmb-2017-0261 29792526

[ref26] Houte S. V. Ekroth A. K. Broniewski J. M. Chabas H. Ashby B. Bondy-Denomy J.. (2016). The diversity-generating benefits of a prokaryotic adaptive immune system. Nature 532, 385–388. doi: 10.1038/NATURE17436, PMID: 27074511 PMC4935084

[ref27] Jamal M. Bukhari S. M. Andleeb S. Ali M. Raza S. Nawaz M. A.. (2019). Bacteriophages: an overview of the control strategies against multiple bacterial infections in different fields. J. Basic Microbiol. 59, 123–133. doi: 10.1002/jobm.201800412, PMID: 30485461

[ref28] Jault P. Leclerc T. Jennes S. Pirnay J. P. Que Y. A. Resch G.. (2019). Efficacy and tolerability of a cocktail of bacteriophages to treat burn wounds infected by *Pseudomonas aeruginosa* (PhagoBurn): a randomised, controlled, double-blind phase 1/2 trial. Lancet Infect. Dis. 19, 35–45. doi: 10.1016/S1473-3099(18)30482-1, PMID: 30292481

[ref29] Joensen K. G. Scheutz F. Lund O. Hasman H. Kaas R. S. Nielsen E. M.. (2014). Real-time whole-genome sequencing for routine typing, surveillance, and outbreak detection of verotoxigenic *Escherichia coli*. J. Clin. Microbiol. 52, 1501–1510. doi: 10.1128/JCM.03617-13, PMID: 24574290 PMC3993690

[ref30] Kalatzis P. G. Castillo D. Katharios P. Middelboe M. (2018). Bacteriophage interactions with marine pathogenic vibrios: implications for phage therapy. Antibiotics 7, 1–23. doi: 10.3390/antibiotics7010015, PMID: 29495270 PMC5872126

[ref9002] Kang C. H. Shin Y. Jang S. Yu H. Kim S. An S.. (2017). Characterization of Vibrio parahaemolyticus isolated from oysters in Korea: resistance to various antibiotics and prevalence of virulence genes. Mar Pollut Bull, 118 261–266. doi: 10.1016/j.marpolbul.2017.02.070 28279505

[ref31] Karunasagar I. Shivu M. M. Girisha S. K. Krohne G. Karunasagar I. (2007). Biocontrol of pathogens in shrimp hatcheries using bacteriophages. Aquaculture 268, 288–292. doi: 10.1016/j.aquaculture.2007.04.049

[ref32] Kim H. W. Hong Y. J. Jo J. I. Ha S. D. Kim S. H. Lee H. J.. (2017). Raw ready-to-eat seafood safety: microbiological quality of the various seafood species available in fishery, hyper and online markets. Lett. Appl. Microbiol. 64, 27–34. doi: 10.1111/lam.12688, PMID: 27747902

[ref33] Kim Y. K. McCarter L. L. (2000). Analysis of the polar flagellar gene system of *Vibrio parahaemolyticus*. J. Bacteriol. 182, 3693–3704. doi: 10.1128/jb.182.13.3693-3704.2000, PMID: 10850984 PMC94540

[ref34] Kortright K. E. Chan B. K. Koff J. L. Turner P. E. (2019). Phage therapy: a renewed approach to combat antibiotic-resistant bacteria. Cell Host Microbe 25, 219–232. doi: 10.1016/j.chom.2019.01.014, PMID: 30763536

[ref35] Le S. Yao X. Lu S. Tan Y. Rao X. Li M.. (2014). Chromosomal DNA deletion confers phage resistance to *Pseudomonas aeruginosa*. Sci. Rep. 4:4738. doi: 10.1038/srep04738, PMID: 24770387 PMC4001099

[ref36] Lefkowitz E. J. Dempsey D. M. Hendrickson R. C. Orton R. J. Siddell S. G. Smith D. B. (2018). Virus taxonomy: the database of the international committee on taxonomy of viruses (ICTV). Nucleic Acids Res. 46, D708–D717. doi: 10.1093/nar/gkx932, PMID: 29040670 PMC5753373

[ref9003] Lesmana M. Subekti D. Simanjuntak C. H. Tjaniadi P. Campbell J. R. Oyofo B. A.. (2001). Vibrio parahaemolyticus associated with cholera-like diarrhea among patients in North Jakarta, Indonesia. Diagn Microbiol Infect Dis, 39 71–75. doi: 10.1016/s0732-8893(00)00232-7 11248518

[ref37] Li H. Durbin R. (2009). Fast and accurate short read alignment with burrows-wheeler transform. Bioinformatics 25, 1754–1760. doi: 10.1093/bioinformatics/btp324, PMID: 19451168 PMC2705234

[ref38] Li C. Shi T. Sun Y. Zhang Y. (2022). A novel method to create efficient phage cocktails via use of phage-resistant bacteria. Appl. Environ. Microbiol. 88:e0232321. doi: 10.1128/aem.02323-21, PMID: 35080902 PMC8939320

[ref39] Li C. Wang Z. Zhao J. Wang L. Xie G. Huang J.. (2021). A novel vibriophage vB_VcaS_HC containing lysogeny-related gene has strong lytic ability against pathogenic bacteria. Virol. Sin. 36, 281–290. doi: 10.1007/s12250-020-00271-w, PMID: 32767211 PMC8087747

[ref40] Liang X. Wang Y. Hong B. Li Y. Ma Y. Wang J. (2022). Isolation and characterization of a lytic *Vibrio parahaemolyticus* phage vB_VpaP_GHSM17 from sewage samples. Viruses 14:1601. doi: 10.3390/v14081601, PMID: 35893666 PMC9331696

[ref41] Lu T. K. Collins J. J. (2007). Dispersing biofilms with engineered enzymatic bacteriophage. Proc. Natl. Acad. Sci. 104, 11197–11202. doi: 10.1073/pnas.0704624104, PMID: 17592147 PMC1899193

[ref42] Lu T. K. Collins J. J. (2009). Engineered bacteriophage targeting gene networks as adjuvants for antibiotic therapy. Proc. Natl. Acad. Sci. 106, 4629–4634. doi: 10.1073/pnas.0800442106, PMID: 19255432 PMC2649960

[ref43] Mangalea M. R. Duerkop B. A. (2020). Fitness trade-offs resulting from bacteriophage resistance potentiate synergistic antibacterial strategies. Infect. Immun. 88, 10–1128. doi: 10.1128/iai.00926-19, PMID: 32094257 PMC7309606

[ref44] Markwitz P. Olszak T. Gula G. Kowalska M. Arabski M. Drulis-Kawa Z. (2021). Emerging phage resistance in *Pseudomonas aeruginosa* PAO1 is accompanied by an enhanced heterogeneity and reduced virulence. Viruses 13:1332. doi: 10.3390/v13071332, PMID: 34372538 PMC8310095

[ref45] Martin J. Schackwitz W. Lipzen A. (2018). Genomic sequence variation analysis by resequencing. Fungal Genom 1775:18. doi: 10.1007/978-1-4939-7804-5_18 29876821

[ref46] Matamp N. Bhat S. G. (2019). Phage endolysins as potential antimicrobials against multidrug resistant vibrio alginolyticus and *Vibrio parahaemolyticus*: current status of research and challenges ahead. Microorganisms 7:84. doi: 10.3390/microorganisms7030084, PMID: 30889831 PMC6463129

[ref47] McArthur A. G. Waglechner N. Nizam F. Yan A. Azad M. A. Baylay A. J.. (2013). The comprehensive antibiotic resistance database. Antimicrob. Agents Chemother. 57, 3348–3357. doi: 10.1128/AAC.00419-13, PMID: 23650175 PMC3697360

[ref48] Moodley A. Kot W. Nälgård S. Jakociune D. Neve H. Hansen L. H.. (2019). Isolation and characterization of bacteriophages active against methicillin-resistant *Staphylococcus pseudintermedius*. Res. Vet. Sci. 122, 81–85. doi: 10.1016/j.rvsc.2018.11.008, PMID: 30468880

[ref49] Nikapitiya C. Chandrarathna H. P. S. U. Dananjaya S. H. S. De Zoysa M. Lee J. (2020). Isolation and characterization of phage (ETP-1) specific to multidrug resistant pathogenic Edwardsiella tarda and its in vivo biocontrol efficacy in zebrafish (*Danio rerio*). Biologicals 63, 14–23. doi: 10.1016/j.biologicals.2019.12.006, PMID: 31889622

[ref50] Nishimura Y. Yoshida T. Kuronishi M. Uehara H. Ogata H. Goto S. (2017). ViPTree: the viral proteomic tree server. Bioinformatics 33, 2379–2380. doi: 10.1093/bioinformatics/btx157, PMID: 28379287

[ref51] North O. I. Brown E. D. (2021). Phage–antibiotic combinations: a promising approach to constrain resistance evolution in bacteria. Ann. N. Y. Acad. Sci. 1496, 23–34. doi: 10.1111/nyas.14533, PMID: 33175408

[ref52] O’Leary N. A. Wright M. W. Brister J. R. Ciufo S. Haddad D. McVeigh R.. (2016). Reference sequence (RefSeq) database at NCBI: current status, taxonomic expansion, and functional annotation. Nucleic Acids Res. 44, D733–D745. doi: 10.1093/nar/gkv1189, PMID: 26553804 PMC4702849

[ref9005] Ottaviani D. Leoni F. Talevi G. Masini L. Santarelli S. Rocchegiani E.. (2013). Extensive investigation of antimicrobial resistance in Vibrio parahaemolyticus from shellfish and clinical sources, Italy. Int J Antimicrob Agents, 42 191–193. doi: 10.1016/j.ijantimicag.2013.05.003 23796895

[ref53] Patro R. Duggal G. Love M. I. Irizarry R. A. Kingsford C. (2017). Salmon provides fast and bias-aware quantification of transcript expression. Nat. Methods 14, 417–419. doi: 10.1038/nmeth.4197, PMID: 28263959 PMC5600148

[ref54] Plaza N. Castillo D. Pérez-Reytor D. Higuera G. García K. Bastías R. (2018). Bacteriophages in the control of pathogenic vibrios. Electron. J. Biotechnol. 31, 24–33. doi: 10.1016/j.ejbt.2017.10.012

[ref55] Pujato S. A. Guglielmotti D. M. Martinez-Garcia M. Quiberoni A. Mojica F. J. (2017). Leuconostoc mesenteroides and *Leuconostoc pseudomesenteroides* bacteriophages: genomics and cross-species host ranges. Int. J. Food Microbiol. 257, 128–137. doi: 10.1016/j.ijfoodmicro.2017.06.009, PMID: 28651078

[ref56] Pujato S. A. Mercanti D. J. Guglielmotti D. M. Rousseau G. M. Moineau S. Reinheimer J. A.. (2015). Phages of dairy *Leuconostoc mesenteroides*: genomics and factors influencing their adsorption. Int. J. Food Microbiol. 201, 58–65. doi: 10.1016/j.ijfoodmicro.2015.02.016, PMID: 25747109

[ref57] Qimron U. Marintcheva B. Tabor S. Richardson C. C. (2006). Genomewide screens for *Escherichia coli* genes affecting growth of T7 bacteriophage. Proc. Natl. Acad. Sci. 103, 19039–19044. doi: 10.1073/pnas.0609428103, PMID: 17135349 PMC1748173

[ref58] Raszl S. M. Froelich B. A. Vieira C. R. W. Blackwood A. D. Noble R. T. (2016). Vibrio parahaemolyticus and *Vibrio vulnificus* in South America: water, seafood and human infections. J. Appl. Microbiol. 121, 1201–1222. doi: 10.1111/jam.13246, PMID: 27459915

[ref59] Ren H. Li Z. Xu Y. Wang L. Li X. (2019). Protective effectiveness of feeding phage cocktails in controlling *Vibrio parahaemolyticus* infection of sea cucumber *Apostichopus japonicus*. Aquaculture 503, 322–329. doi: 10.1016/j.aquaculture.2019.01.006

[ref60] Robinson M. D. McCarthy D. J. Smyth G. K. (2010). edgeR: a bioconductor package for differential expression analysis of digital gene expression data. Bioinformatics 26, 139–140. doi: 10.1093/bioinformatics/btp616, PMID: 19910308 PMC2796818

[ref61] Rong R. Lin H. Wang J. Khan M. N. Li M. (2014). Reductions of *Vibrio parahaemolyticus* in oysters after bacteriophage application during depuration. Aquaculture 418-419, 171–176. doi: 10.1016/j.aquaculture.2013.09.028

[ref62] Ruohan W. Xianglilan Z. Jianping W. Shuai Cheng L. I. (2022). DeepHost: phage host prediction with convolutional neural network. Brief. Bioinform. 23:385. doi: 10.1093/bib/bbab385, PMID: 34553750

[ref63] Samson J. E. Magadán A. H. Sabri M. Moineau S. (2013). Revenge of the phages: defeating bacterial defences. Nat. Rev. Microbiol. 11, 675–687. doi: 10.1038/nrmicro3096, PMID: 23979432

[ref64] Sar N. E. C. H. E. M. I. A. McCARTER L. I. N. D. A. Simon M. E. L. V. I. N. Silverman M. I. C. H. A. E. L. (1990). Chemotactic control of the two flagellar systems of *Vibrio parahaemolyticus*. J. Bacteriol. 172, 334–341. doi: 10.1128/jb.172.1.334-341.1990, PMID: 2294089 PMC208437

[ref65] Sharma S. Chatterjee S. Datta S. Prasad R. Dubey D. Prasad R. K.. (2017). Bacteriophages and its applications: an overview. Folia Microbiol. 62, 17–55. doi: 10.1007/s12223-016-0471-x 27718043

[ref66] Shen Y. Loessner M. J. (2021). Beyond antibacterials–exploring bacteriophages as antivirulence agents. Curr. Opin. Biotechnol. 68, 166–173. doi: 10.1016/j.copbio.2020.11.004, PMID: 33333352

[ref67] Stalin N. Srinivasan P. (2017). Efficacy of potential phage cocktails against Vibrio harveyi and closely related vibrio species isolated from shrimp aquaculture environment in the south east coast of India. Vet. Microbiol. 207, 83–96. doi: 10.1016/j.vetmic.2017.06.006, PMID: 28757045

[ref68] Steinegger M. Söding J. (2017). MMseqs2 enables sensitive protein sequence searching for the analysis of massive data sets. Nat. Biotechnol. 35, 1026–1028. doi: 10.1038/nbt.3988, PMID: 29035372

[ref69] Sulakvelidze A. Alavidze Z. Morris J. G. Jr. (2001). Bacteriophage therapy. Antimicrob. Agents Chemother. 45, 649–659. doi: 10.1128/aac.45.3.649-659.2001, PMID: 11181338 PMC90351

[ref70] Tang Y. Li J. Wang Y. Song Z. Ying H. Kong L.. (2022). *Campylobacter jejuni* developed the resistance to bacteriophage CP39 by phase variable expression of 06875 encoding the CGPTase. Viruses 14:485. doi: 10.3390/v14030485, PMID: 35336892 PMC8949473

[ref9004] Wang J. Zhao F. Sun H. Wang Q. Zhang C. Liu W.. (2019). Isolation and characterization of the Staphylococcus aureus bacteriophage vB_SauS_SA2. AIMS Microbiol. doi: 10.3934/microbiol.2019.3.285 PMC6787349 31663062

[ref71] Wang X. Loh B. Gordillo Altamirano F. Yu Y. Hua X. Leptihn S. (2021). Colistin-phage combinations decrease antibiotic resistance in *Acinetobacter baumannii* via changes in envelope architecture. Emerg Microbes Infect 10, 2205–2219. doi: 10.1080/22221751.2021.2002671, PMID: 34736365 PMC8648044

[ref72] Wang R. Ng Y. K. Zhang X. Wang J. Li S. C. (2022). A graph representation of gapped patterns in phage sequences for graph convolutional network. bioRxiv [Preprint], 2022.

[ref73] Westra E. R. van Houte S. Oyesiku-Blakemore S. Makin B. Broniewski J. M. Best A.. (2015). Parasite exposure drives selective evolution of constitutive versus inducible defense. Curr. Biol. 25, 1043–1049. doi: 10.1016/j.cub.2015.01.065, PMID: 25772450

[ref74] Williams S. L. Jensen R. V. Kuhn D. D. Stevens A. M. (2017). Analyzing the metabolic capabilities of a *Vibrio parahaemolyticus* strain that causes early mortality syndrome in shrimp. Aquaculture 476, 44–48. doi: 10.1016/j.aquaculture.2017.03.030

[ref75] Yang Y. Shen W. Zhong Q. Chen Q. He X. Baker J. L.. (2020). Development of a bacteriophage cocktail to constrain the emergence of phageresistant *Pseudomonas aeruginosa*. Front. Microbiol. 11:327. doi: 10.3389/fmicb.2020.00327, PMID: 32194532 PMC7065532

[ref76] Yang Z. Yin S. Li G. Wang J. Huang G. Jiang B.. (2019). Global transcriptomic analysis of the interactions between phage φAbp1 and extensively drug-resistant *acinetobacter baumannii*. mSystems 4, 10–1128. doi: 10.1128/mSystems.00068-19, PMID: 31020041 PMC6469957

[ref77] Zankari E. Hasman H. Cosentino S. Vestergaard M. Rasmussen S. Lund O.. (2012). Identification of acquired antimicrobial resistance genes. J. Antimicrob. Chemother. 67, 2640–2644. doi: 10.1093/jac/dks261, PMID: 22782487 PMC3468078

[ref79] Zhang M. Qian J. Xu X. Ahmed T. Yang Y. Yan C.. (2022). Resistance of *Xanthomonas oryzae* pv. Oryzae to lytic phage X2 by spontaneous mutation of lipopolysaccharide synthesis-related glycosyltransferase. Viruses 14:1088. doi: 10.3390/v14051088, PMID: 35632829 PMC9143033

[ref80] Zhang X. Wang R. Xie X. Hu Y. Wang J. Sun Q.. (2022). Mining bacterial NGS data vastly expands the complete genomes of temperate phages. NAR Genom Bioinform. 4:57. doi: 10.1093/nargab/lqac057, PMID: 35937545 PMC9346568

