# Corrigendum: The trade-off of *Vibrio parahaemolyticus* between bacteriophage resistance and growth competitiveness

**DOI:** 10.3389/fmicb.2024.1459985

**Published:** 2024-08-16

**Authors:** Xiuxiu Zeng, Shanyan Liang, Jiayi Dong, Guosheng Gao, Yaoren Hu, Yuechao Sun

**Affiliations:** ^1^Ningbo No.2 Hospital, Ningbo, Zhejiang, China; ^2^Guoke Ningbo Life Science and Health Industry Research Institute, Ningbo, Zhejiang, China

**Keywords:** vibriophage, bacteriophage therapy, anti-phage mutant, bacteriophage resistance, trade-off

In the published article, there was an error in affiliation(s) (1 and 2). Instead of “(1. Guoke Ningbo Life Science and Health Industry Research Institute, Ningbo, Zhejiang, China; 2. Ningbo Hospital, Ningbo, Zhejiang, China)”, it should be “(1. Ningbo No.2 Hospital, Ningbo, Zhejiang, China; 2. Guoke Ningbo Life Science and Health Industry Research Institute, Ningbo, Zhejiang, China)”.

In the published article, there was an error regarding the affiliation(s) for (Xiuxiu Zeng). As well as having affiliation(s) (2. Guoke Ningbo Life Science and Health Industry Research Institute, Ningbo, Zhejiang, China), they should also have (1. Ningbo No.2 Hospital, Ningbo, Zhejiang, China).

In the published article, there was an error in (Figure 1E) as published (latest image results has not been updated). The corrected (Figure 1E) and its caption appear below.

**Figure 1:**
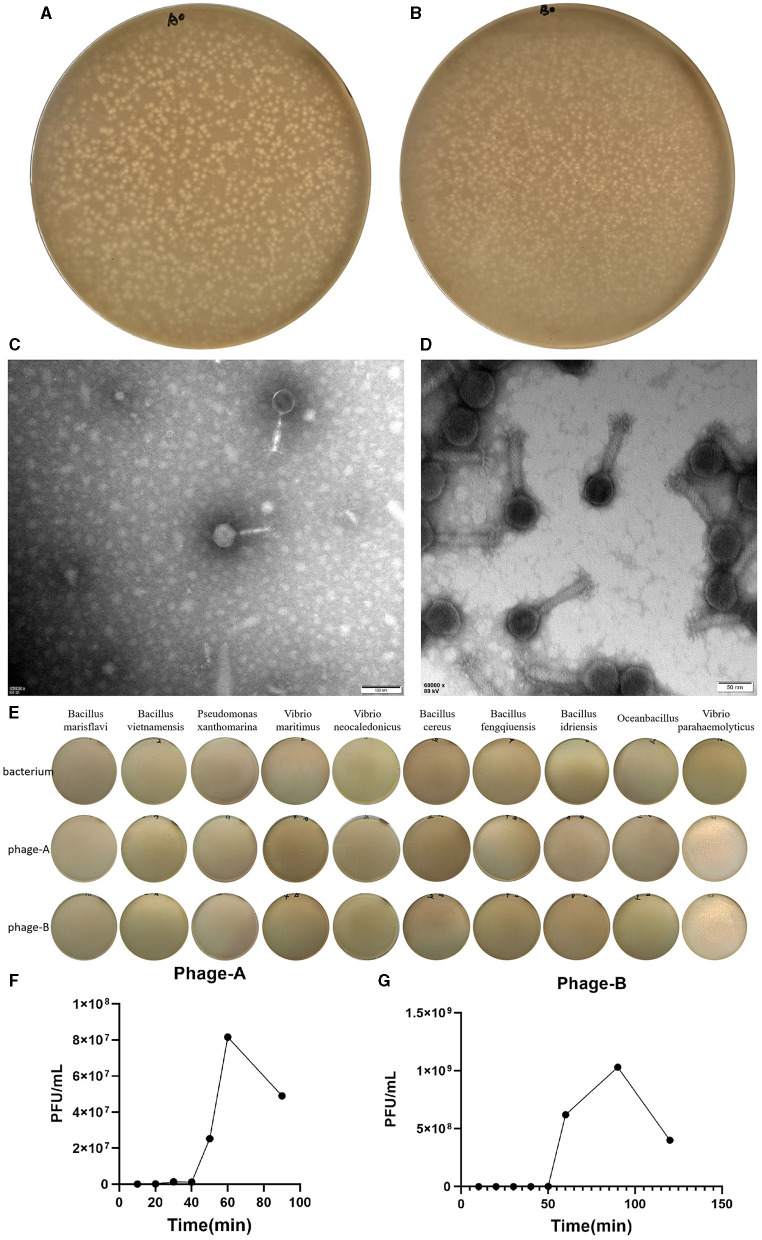
Characterization of Bacteriophage. **(A)** Plaques of bacteriophage vB_VpaS_PGA. **(B)** Plaques of bacteriophage vB_VpaS_PGB. **(C)** Transmission electron microscopy of bacteriophage vB_VpaS_PGA. **(D)** Transmission electron microscopy of bacteriophage vB_VpaS_PGB. **(E)** Host range of bacteriophage vB_VpaS_PGA and vB_VpaS_PGB. **(F)** One-step growth curve of PGA. **(G)** One-step growth curve of PGB.

In the published article, there was an error in the Funding statement. There are two fund projects missing. The correct Funding statement appears below.

