# Supplementary figures and images for: The trade-off of Vibrio parahaemolyticus between bacteriophage resistance and growth competitiveness
Source: Front Microbiol. 2024 Jun 11;15:1346251. doi: 10.3389/fmicb.2024.1346251 (PMC11196418; doi:10.3389/fmicb.2024.1346251)

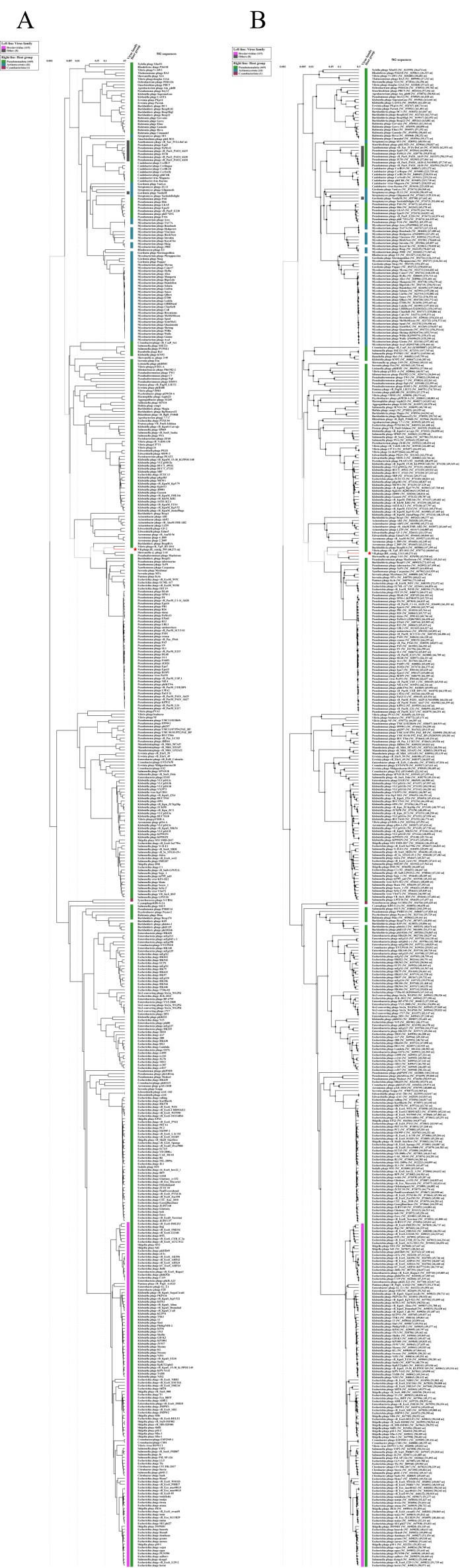

Supplement: Supplementary file 7 [file Image_1.jpg]
